# Supplementary material for: Poisoning among children in Malaysia: A 10-years retrospective study
Source: PLoS One. 2022 Apr 28;17(4):e0266767. doi: 10.1371/journal.pone.0266767 (PMC9049302; doi:10.1371/journal.pone.0266767)
Supplement: Attachment — Submitted filename: Response to Reviewers.docx [file pone.0266767.s001.docx]

**Response to Reviewers’ Comments**

Article ref. no: PONE-D-21-32551

Title: Poisoning Among Children in Malaysia: A 10-Year Review

**Dear Editor:**

On behalf of all the authors, I would like to thank all the reviewers for their deep and thorough review and the constructive comments on our submitted manuscript.

All the comments made by the reviewers have helped in enhancing the quality and clarity of the manuscript. We have also included below a letter detailing our responses to the comments of the reviewers.

Based on the reviewers’ comments, we have incorporated all the necessary changes. Changes made in our responses to the comments are highlighted in the Track Changes version of the revised manuscript. I hope this revised manuscript has met the level of their satisfaction.

I am very grateful to you and the reviewers for the time spent on this manuscript.

We look forward to receiving your favorable response.

Sincerely,

The Corresponding Author

**Iqdam Abdulmaged Alwan**

M. Sc. (Health Toxicology),

Integrative Medicine Cluster, Advanced Medical and Dental Institute, Universiti Sains Malaysia, Kepala Batas, Penang, Malaysia.

**Response to Referee #1 Comments**

**Comment 1:**

Title of the study is “Poisoning Among Children in Malaysia: A 10-Year Review”. Out of the five key words three are a repeat from the title. It is suggested to authors to replace these three repeated keywords, to increase the searchability of the article over internet, if published. The keywords should preferably from the MeSH Vocabulary database.

**Response:**

The authors are grateful for the constructive comment and suggestion to improve the clarity of the study. We have considered the reviewer’s suggestions, new keywords were extracted from the MeSH Vocabulary database as per requested. Please see the keywords in the revised manuscript.

**Comment 2:**

The introduction part should be combined in form of two or three paragraphs.

**Response:**

We agreed with the reviewer suggestion. The parts of the introduction were combined into three paragraphs. Please see the introduction part in the revised manuscript.

**Comment 3:**

The vancouver style should be used for references.

**Response:**

All the references were made in the Vancouver style in the revised manuscript as the reviewer advised.

**Comment 4:**

The manuscript requires the following grammatical corrections:

**Response:**

We would like to thank the reviewer for helping us enhancing the quality and clarity of the manuscript.

**Comment 4.1:**

Line 37-38: “Poisoning commonly occurs among children as a consequence of their curiosity, where they have a tendency to explore and investigate their surroundings.” Should be replaced by “Poisoning commonly occurs among children due to their curiosity, where they tend to explore and investigate their surroundings.”

**Response:**

The paragraph was rephrased as the reviewer suggested. Please see the revised manuscript, line: 25, 26.

**Comment 4.2:**

Line 76-78: “Based on statistics by the World Health Organization (WHO), acute poisoning is responsible for more than 45,000 deaths every year among children and youths below the age of 20 years” should be replaced by “Based on the World Health Organization (WHO) statistics, acute poisoning is responsible for more than 45,000 deaths every year among children and youths below the age of 20 years.”

**Response:**

The sentence has been replaced in the revised manuscript as the reviewer suggested. Please see the revised manuscript, line: 63-65.

**Comment 4.3:**

Line 91: Remove comma after Although

**Response:**

The sentence has been corrected in the revised manuscript. Line: 78.

**Comment 4.4:**

Line 96-100: Replace “DPIS is the main source of information on poisoning risks and toxicity, and it serves healthcare professionals and the general populace with the relevant information when they enquire about such matters. The NPC operates 24/7, whereby pharmacists, who are trained in toxicology and who specialize in providing drug and poison information services, offer advice to enquirers, usually via telephone calls.”

With

“DPIS is the main source of information on poisoning risks and toxicity. It serves healthcare professionals and the general populace with the relevant information when they inquire about such matters. The NPC operates 24/7, whereby pharmacists, trained in toxicology and specialize in providing drug and poison information services, offer advice to enquirers, usually via telephone calls.”

**Response:**

The sentence has been replaced in the revised manuscript, line: 83-87.

**Comment 4.5:**

Line 131: Replace “Ethic” with “Ethics”

**Response:** Thank you for pointing out this typo, the word “Ethic” has been replaced with “Ethics” as the reviewer suggested. Please see the revised manuscript, line: 121.

**Comment 4.6:**

Line 144: Replace “increased constantly” with “constantly increased”

**Response:**

The sentence has been corrected as the reviewer suggested. Please see the revised manuscript, line: 132.

**Comment 4.7:**

Line 252: Remove “in nature”

**Response:**

The word “in nature” has been removed as the reviewer suggested. Please see the revised manuscript, line: 217.

**Comment 4.8:**

Line 256: ‘This’ is an unclear antecedent. Please rectify.

**Response:**

The paragraph was rephrased and rectified as the reviewer suggested. Please see the revised manuscript, line: 221.

**Comment 4.9:**

Line 323-325: Replace “Appropriate strategies and legislations should be implemented to regulate the sale of over-the-counter medications and to ensure that potentially dangerous chemicals are sold in child-resistant containers.” with “Appropriate strategies and legislation should be implemented to regulate the sale of over-the-counter medications and ensure that potentially dangerous chemicals are sold in child-resistant containers.”

**Response:**

The sentence has been corrected in the conclusion section. Please see the revised manuscript, line: 290-292.

**Response to referee #2 comments**

**Comment 1:**

Title can be changed to Poisoning Among Children in Malaysia: A 10-years retrospective study.

**Response:**

The authors are grateful for the reviewer’s suggestion that improves the description of the title. The title has been changed in the revised version. Please see the revised manuscript.

**Comment 2:**

The standard definition of 'child' given by WHO or UNESCO should be included.

**Response:**

The standard definition of 'child' given by UNESCO has been included in the method section. Please see the revised manuscript, line: 109-111.

**Comment 4:**

In the abstract, you have mentioned the inclusion of samples of age 19 years and below, whereas, in the methodology of the manuscript, you have mentioned the samples of age 18 years and below. Please clarify the disparity.

**Response:**

We would like to thank the reviewer for highlighting this matter effectively; we have rectified all the mistakes relating to the inclusion of samples age. Please see the revised manuscript.

**Comment 5:**

Also, you have subdivided the age groups as below 6 years, 6 to 12 years and above 12 years. Table 1 has categories of 0 to 5 years, 6 to 12 years and 13 to 18 years. Please maintain uniformity.

**Response:**

We would like to thank the reviewer once again for pointing out this issue, we apologize for this error in the age categories; we have corrected all the mistakes related to this issue accordingly, please refer to the revised manuscript.

**Comment 6:**

Also, clarify the reason to subdivide the age group into these three categories.

**Response:**

Authors are grateful to the reviewer for highlighting this point. This classification of age is consistent with the reports of the American Association of Poison Control Centers’ (NPDS). Please see the revised manuscript, line: 112-113.

**Comment 7:**

Table 1 can be subdivided into more tables as extensive information has been clustered, which is confusing.

**Response:**

We thank the reviewer for the constructive suggestion to improve the clarity. However, we believe that the table should not be subdivided into more tables; we would like to keep all information about (gender, incident type, route and place of exposure) in one table that would make it easier to the reader to find the data.

**Comment 8:**

In table number 1, 'character' can be replaced by ‘the number of cases’; 'Incident type' can be replaced by 'the manner of poisoning'.

**Response:**

We have replaced 'character' with ‘the number of cases’; 'Incident type' with 'the manner of poisoning'. Please see the revised manuscript, table 1, line: 158.

**Comment 9:**

In table number 1, the other category in route of exposure should be subdivided in the table only.

**Response:**

We thank the reviewer for this suggestion. In the table number 1, we have listed only the main 3 categories in each section, which represent more than 95% of the total number. While the other categories were non-significant compared to the main three categories (less than 5%), thus, we specified them in the table footnote.

**Comment 10:**

Please specify the reasons for gender remaining unknown in about 2000 cases.

**Response:**

**T**he authors agree with the reviewer comment. The unclassified gender category marked as (unknown) mostly related to individuals aged 0 to 5 years old without a confirmed record in the form of gender was obtained from the caller. We have added this information as a limitation of this study in the discussion section, line: 273-275, in the revised manuscript.

**Comment 11:**

Legal scenarios in Malaysia pertaining to various poisoning agents and therapeutic agents can be discussed.

**Response:**

The legal scenarios in Malaysia relating to different poisoning agents were discussed in the discussion section as per the reviewer suggestion. Please see the revised manuscript, line: 236-246.

**Comment 12:**

The statistical analysis should have been performed appropriately and rigorously.

**Response:**

Authors are grateful to the reviewer for his positive and encouraging comments, however, as far as we believe that statistical analysis was not needed in this study, since the primary objective of this study is to highlight the trends in children poisoning inquiries, descriptive analysis was adequate for data presentation in this case.
